# Injectable polyethylene glycol-fibrinogen hydrogel adjuvant improves survival and differentiation of transplanted mesoangioblasts in acute and chronic skeletal-muscle degeneration

**DOI:** 10.1186/2044-5040-2-24

**Published:** 2012-11-26

**Authors:** Claudia Fuoco, Maria Lavinia Salvatori, Antonella Biondo, Keren Shapira-Schweitzer, Sabrina Santoleri, Stefania Antonini, Sergio Bernardini, Francesco Saverio Tedesco, Stefano Cannata, Dror Seliktar, Giulio Cossu, Cesare Gargioli

**Affiliations:** 1 Department of Biology, Tor Vergata Rome University, Rome, Italy; 2 Division of Regenerative Medicine, San Raffaele Scientific Institute, Milan, Italy; 3 Faculty of Biomedical Engineering, Technion – Israel Institute of Technology, Haifa, Israel; 4 Department of Cell and Developmental Biology, UCL, London, UK; 5 IRCCS MultiMedica, Milan, Italy

**Keywords:** Stem cells, Mesoangioblasts, Hydrogel, Muscular dystrophy, Muscle regeneration, Cell therapy, Tissue engineering

## Abstract

**Background:**

Cell-transplantation therapies have attracted attention as treatments for skeletal-muscle disorders; however, such research has been severely limited by poor cell survival. Tissue engineering offers a potential solution to this problem by providing biomaterial adjuvants that improve survival and engraftment of donor cells.

**Methods:**

In this study, we investigated the use of intra-muscular transplantation of mesoangioblasts (vessel-associated progenitor cells), delivered with an injectable hydrogel biomaterial directly into the tibialis anterior (TA) muscle of acutely injured or dystrophic mice. The hydrogel cell carrier, made from a polyethylene glycol-fibrinogen (PF) matrix, is polymerized *in situ* together with mesoangioblasts to form a resorbable cellularized implant.

**Results:**

Mice treated with PF and mesoangioblasts showed enhanced cell engraftment as a result of increased survival and differentiation compared with the same cell population injected in aqueous saline solution.

**Conclusion:**

Both PF and mesoangioblasts are currently undergoing separate clinical trials: their combined use may increase chances of efficacy for localized disorders of skeletal muscle.

## Background

Skeletal muscles are primarily responsible for controlling voluntary movement and posture. They can self-repair in response to moderate injuries, but are not able to regenerate when significant loss of tissue occurs in extensive trauma or surgery. Similarly, they cannot sustain repeated cycles of degeneration/regeneration, such as occurs in severe forms of muscular dystrophy [1], which are difficult diseases to treat. Such conditions affect the large majority of skeletal muscles, which are composed of large multinucleated post-mitotic fibers surrounded by a thick basal lamina. Delivery of cells or vectors into these muscles still represents a significant challenge [1]. Reconstructive strategies, such as autologous muscle transplantation and intra-muscular injection of progenitor cells yield only modest therapeutic outcomes, mainly because the tissue often presents an inflamed or sclerotic environment that results in poor survival and only modest integration of engrafted cells, and the cells are also targets of an immune reaction [2-5]. Moreover, the *in vitro* cultivation history of the grafted cells can also negatively affect the efficacy of myoblast transplantation, although this may be prevented by culturing cells on soft hydrogels [6]. Among the new therapeutic strategies for treating muscular dystrophies, stem-cell transplantation is becoming a promising clinical option [7]. Systemic injections of vessel-associated progenitor cells called mesoangioblasts, which overcome some of the problems associated with myoblast intra-muscular injections, has been shown to result in better long-term survival of donor cells, and in partial restoration of muscle structure and function in dystrophic mice [8,9] and dogs [10]. The efficacy of mesoangioblasts is mainly due to their ability to cross the endothelium and to migrate extensively in the interstitial space, where they are recruited by regenerating muscle to reconstitute new functional myofibers. Consequently, a phase I/II clinical trial based on intra-arterial delivery of donor-derived mesoangioblasts is currently ongoing in children affected by Duchene Muscular Dystrophy at the San Raffaele Hospital in Milan (EudraCT no. 2011-000176-33).

A completely different approach using cell transplantation (that is, tissue engineering), may be useful for whole-muscle reconstruction after severe damage caused by traumatic injury or surgical ablation [11,12]. Tissue engineering uses two main components: the cells themselves, and biomaterials in which the cells are embedded [11]. To support optimal *in vivo* muscle differentiation, the biomaterials should possess characteristics such as bioactivity, cell-mediated biodegradability, minimal cytotoxicity, and controllable properties including stiffness [13]. With these issues in mind, natural components of the extracellular matrix (ECM) have been reconstituted as biomaterials that mimic the microenvironment of skeletal muscle and thus support better regeneration.

Many different polymers, of both natural and synthetic origins, have previously been used as scaffolds for the regeneration of skeletal and cardiac muscle. In cardiac repair, for example, many scaffolds have been tested in animal trials with rats and dogs, but very few are being tested in human clinical trials [14,15]. Nevertheless, compared with direct myocardial injection of cells alone, it is strikingly clear that tissue-engineering strategies offer better pre-clinical results, including augmenting the engrafted cardiomyocyte population and improving the contractile function of the ischemic heart [16]. Likewise, in the field of skeletal-muscle regeneration, Rossi and colleagues reported similarly good results with biomaterials and tissue engineering. These authors used freshly isolated myoblasts and hyaluronic acid ester-based hydrogels, polymerized *in situ*, to promote improved reconstruction of a partially ablated skeletal-muscle injury [17].

In the current investigation, we evaluated an approach based upon local delivery of mesoangioblasts that was facilitated by a semi-synthetic hydrogel made from polyethylene glycol (PEG) and fibrinogen. This PEG-fibrinogen (PF) hydrogel has a proven track record in three-dimensional cell culture, in cardiac cell therapy and tissue engineering [18]. One advantage of the PF hydrogel is its ability to undergo controlled and localized liquid-to-solid transition (gelation) in the presence of a cell suspension inside a muscle injury. Another very important feature of the PF hydrogel is its chemical composition; the PEG enables control over the material properties and the fibrinogen provides inherent bioactivity, including cell-adhesion motifs and protease-degradation sites [19]. We tested different PF formulations, embedding mesoangioblasts within them and injecting the grafts into acutely injured muscle and also into dystrophic muscle at an advanced stage of the disease, in order to evaluate the ability of the PF cell carrier to improve the therapeutic effect of donor mesoangioblasts.

## Methods

### Animal procedures

Ethics approval for the animal experiments was obtained from the Italian Ministry of Health (protocol #163/2011-B; released on 16 September 2011) and all experiments were conducted in accordance with the rules of good animal experimentation (IACUC, number 432, dated 12 March 2006).

### Preparation of mesoangioblasts and culture conditions

Mesoangioblasts were cultured at 37°C (5% CO2) in petri dishes with DMEM (Dulbecco’s modified Eagle’s medium with GlutaMAX; Gibco-BRL,Gaithersburg, MD, USA), supplemented with heat-inactivated 10% FCS (EuroClone), 100 IU/ml penicillin and 100 mg/ml streptomycin [20]. Mesoangioblasts were transduced with third-generation lentiviral vectors encoding the nuclear β-Galactosidase. and mesoangioblasts expressing nuclear lacZ (nlacZ-mesoangioblasts) were cultured and used for *in vitro* differentiation or intra-muscular injection [8].

### Polyethylene glycol-fibrinogen

PEG-fibrinogen was produced and polymerized as described previously [19]. Briefly, PEG-fibrinogen was prepared at a desired concentration and diluted with sterile PBS as required. A photoinitiator (Igracure™ 2959; Ciba Specialty Chemicals, USA) was added to the PEG-fibrinogen mixture at a final concentration of 0.1% w/v. Cells were added at the desired concentration and the solution was immediately exposed to UV light (365 nm, 4–5 mW/cm^2^) for 5 minutes for the *in vitro* experiments. *In vivo* experiments were exposed to UV light (365 nm, 200 mW/cm^2^) using a hand-held light gun (LED-200; Electro-lite Corp., Bethel, CT USA) for 1 minute.

### Animals and treatments

Rag2 γ-chain null mice (4 months old) and α-sarcoglycan knockout/severe combined immunodeficiency beige (α-SGKO/SCIDbg) mice [21] (12 months old) were used for intra-muscular injection. Briefly, mice were anesthetized with an intra-muscular injection of physiologic saline 10 ml/kg containing ketamine 5 mg/ml and xylazine 1 mg/ml. For the liquid nitrogen (N_2_) muscle-crush injury, a small skin incision was made over the tibialis anterior (TA) muscle of anesthetized mice. A liquid-nitrogen-cooled needle (0.20 mm diameter) was inserted along the craniocaudal axis of the TA twice, 30 seconds for each insertion. For intra-muscular cell delivery, approximately 3 × 10^5^ nlacZ-mesoangioblasts were injected into the TA via a 0.20 mm diameter needle inserted along the craniocaudal axis of the muscle. For PF-embedded nlacZ-mesoangioblast injections, a limited incision was made on the medial side of the leg to separate the TA from the skin and to allow *in vivo* PF photopolymerization. A subgroup of animals was injected intraperitoneally with 5-bromo-2-deoxyuridine (BrdU) 100 mg/kg (RPN 20; GE Healthcare, Princeton, NJ, USA) to label proliferating cells 2 hours after mesoangioblast transplantation. The BrdU-labeled mice were killed 48 hours after cell injection.

### Cell apoptosis

The presence of apoptotic cells was examined using terminal deoxynucleotidyl transferase dUTP nick-end labeling (TUNEL) staining (Roche Diagnostics, Basel, Switzerland) in 10 μm cryosections. Positive control sections were treated with DNaseI (Roche Diagnostics, Basel, Switzerland) for 20 minutes at 37°C. Sections were incubated with the TUNEL reagent at 37°C for 30 minutes before being counterstained with 4,6-diamidino-2-phenylindole (DAPI).

### Immunohistochemistry

The tissue samples were fixed in 4% paraformaldehyde for 30 minutes at 4°C and washed in PBS, embedded in optimal cutting temperature compound, and flash-frozen in liquid-nitrogen-cooled isopentane. Sections were cut at a thickness of 8 μm on a cryostat (Leica, Heerbrugg, Switzerland) and washed with buffer (PBS containing 0.2% Triton X-100). The sections were then incubated with primary antibody (rabbit anti-laminin; Sigma-Aldrich, St Louis, MO, USA) diluted to a final concentration of 1:100 with blocking buffer (PBS containing 0.2% Triton X-100 and 20% heat-inactivated goat serum) for 20 minutes at room temperature. Sections were washed with washing solution (PBS containing 0.2% Triton X-100 and 1% BSA), and then incubated with the secondary antibody (horseradish peroxidase-conjugated goat anti-rabbit; Chemicon International Inc., Temecula, CA, USA), diluted 1:500 in 20% goat serum. The immune reaction was developed using 3-amino-9 ethylcarbazole substrate (AEC; Sigma-Aldrich).

Afterwards, the sections were stained with X-Gal to reveal β-galactosidase-positive cells as described previously [22]. Briefly, the sections were washed twice with PBS for 5 minutes each and incubated for 24 hours at 37°C with an X-Gal working solution. This solution is composed of the X-Gal stock solution (X-Gal 40 mg/ml in N,N-dimethyl formamide, which was stored at −20°C and protected from light) diluted 1 in 40 in X-Gal dilution buffer (crystalline potassium ferricyanide 5 mmol/l, potassium ferricyanide trihydrate 5 mmol/l, and magnesium chloride 2 mmol/l in PBS, which was protected from light, and stored at 4°C). Sections were washed twice with PBS for 5–10 minutes each, and then covered directly with aqueous mounting medium (Aqua Poly/Mount; Polysciences Inc., Warrington, PA, USA) The lacZ-positive nuclei were counted in five randomly selected fields of three different non-adjacent transverse sections from the largest TA portion taken from three mice per experimental group.

### Immunofluorescence experiments

Immunofluorescence procedures were performed essentially as described previously [22]. Briefly, the specimens were prepared as described above, and then incubated with primary antibodies diluted with blocking buffer for 20 minutes at room temperature. The primary antibodies used were: mouse anti-α-SG (Ad1/20A6; Vector Laboratories Inc., Burlingame, CA, USA) 1:100 dilution, rabbit anti-laminin (#9393; Sigma-Aldrich) at 1:500, rabbit anti-lacZ (Cappel Laboratories, Durham, NC, USA) 1:100, mouse anti-Pax7 and anti-Myosin Heavy Chain (MF20) (Developmental Studies Hybridoma Bank, Iowa City, IA, USA) 1:100. After several washes with buffer, sections were incubated with secondary antibodies diluted with blocking buffer for 1 hour at room temperature. The secondary antibodies (all used at 1:500) were anti-mouse FITC (Chemicon International Inc.), anti-rabbit Alexa488, and anti-rat Alexa488 (both Molecular Probes, Eugene, OR, USA). Sections were counterstained with DAPI to detect nuclei, washed several times with wash buffer, and mounted (Vectorshield; Vector Laboratories Inc.). To visualize BrdU, a commercial kit was used, and sections were treated with nuclease/anti-BrdU solution provided in the kit (RPN20, GE Healthcare, Princeton, NJ, USA) for 1 hour at room temperature in accordance with the manufacturer’s instructions. Sections were washed three times in PBS, and incubated for 30 minutes at room temperature with Alexa Fluor 488 secondary antibody against mouse (Molecular Probes). Sections were counterstained with 4^′^,6-diamidino-2-phenylindole (DAPI), washed in PBS, and mounted as described above.

### Immunoblotting

Tissue samples (n = 3 for each time point per group) of TA treated with PF-embedded mesoangioblasts from α-SG null mice were homogenized in liquid nitrogen, mixed with lysis buffer (50 mmol/l Tris/HCl, pH 7.4, 1 mmol/l EDTA, 1 mmol/l EGTA, 1% Triton X-100, 1 mmol/l), and protease inhibitor cocktail (Sigma-Aldrich), and separated by centrifugation at 12,000 *g* for 10 minutes at 4°C to remove the nuclei and cellular debris. Protein concentrations were determined by bicinchoninic acid (BCA) protein assay (Pierce Biotechnology Inc., Rockford, IL, USA) using BSA as a standard. Total homogenates were separated by SDS-PAGE. For western blotting analysis, proteins were transferred to membranes (Immobilon; Amersham Biosciences Inc., Piscataway, NJ, USA), saturated with blocking solution (1% BSA and 0.1% Tween-20 (Sigma-Aldrich) in PBS) and hybridized with cleaved caspase-3 rabbit monoclonal antibody (#9669; Cell Signaling Technology, Danvers, MA, USA), α-SG mouse monoclonal antibody (Ad1/20A6; Vector Laboratories) or lacZ polyclonal antibody (#55976; Cappel Laboratories) at 1:1,000 dilution, or with GAPDH monoclonal antibody (GAPDH-71.1; Sigma-Aldrich) at 1:10,000 dilution for 1 hour at room temperature. The blots were washed three times (15 minutes each at room temperature) with blocking solution, and then reacted with anti-mouse or anti-rabbit secondary antibody conjugated with HRP (Bio-Rad Laboratories, Inc., Hercules, CA, USA) at 1:3,000 dilution for 1 hour at room temperature. The blots were then washed three times, and finally visualized with an enhanced chemiluminescent immunoblotting detection system (Pierce Biotechnology Inc).

### Statistical analysis

Statistical significance of the differences between means was assessed by one-way analysis of variance (ANOVA) followed by the Student-Newman-Keuls test to determine which groups were significantly different from the others. When only two groups had to be compared, the unpaired Student’s *t*-test was used. *P*<0.05 was considered significant. Values are expressed as means ± standard deviation (SD).

## Results

### Polyethylene glycol-fibrinogen ameliorates *in vitro* muscle differentiation of mesoangioblasts

**Figure 1:**
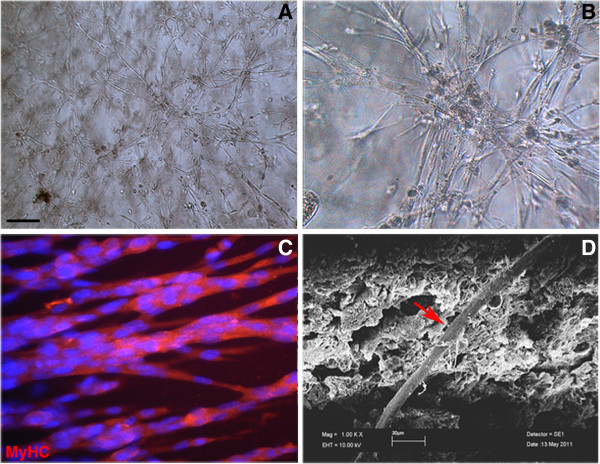
**Mesoangioblasts cultured in polyethylene glycol-fibrinogen (PF) hydrogels.** (**A**,**B**) Phase-contrast images of mesoangioblasts in 8 mg/ml PF hydrogel, giving rise to a robust three-dimensional myofiber network. (**C**) Immunofluorescence showing multinucleated muscle fibers; staining is with an antibody against myosin heavy chain (MyHC; red) and nuclei counterstaining with 4,6-diamidino-2-phenylindole (DAPI; blue). (**D**) Scanning electron microscopy image revealing the presence of differentiating skeletal-muscle fibers (red arrows) within the PF hydrogel. Scale bar: (**A**) 200 μm, (**B**) 50 μm and (**C**) 10 μm.

Initially different hydrogels [23-25] and different myogenic cells were tested to assess different combinations of scaffold and cell that would promote muscle differentiation *in vitro*. The different cells tested exhibited good differentiation capabilities when cultured in PF hydrogel [19] compared with other biomaterials such as fibrin or TG-PEG (see Additional file 1: Figure S1). For the present work, we choose mesoangioblasts (vessel-associated mesoderm progenitors that are distinct from satellite cells, but are still able to undergo robust myogenesis *in vivo* and *in vitro*, and that are currently in phase I/II clinical trials [22,26,27]), as our myogenic stem/progenitor cell. We used these to evaluate the influence of PF on skeletal muscle cell differentiation, and to evaluate the possibility of using mesoangioblasts plus PF as a combination approach for translational clinical applications. Mesoangioblasts, together with a PF formulation that results in a matrix with a stiffness that has been optimized for muscle differentiation [28], were tested prior to our *in vivo* experiments, using different concentrations of the PF precursor ranging from 4 to 12 mg/ml; the optimal composition in terms of cell attachment and myogenic differentiation was found to be 8 mg/ml (see Additional file 1: Figure S2). As part of our *in vitro* testing, the mesoangioblasts were transduced with a lentiviral vector expressing nuclear β-galactosidase (nlacZ-mesoangioblasts) for easier tracking. The nlacZ-mesoangioblasts (3 × 10^5^) were suspended in PF precursor solution (8 mg/ml) and cast in silicone moulds by photopolymerization. Three days after gelation in regular culture, the PF constructs exhibited a homogeneous distribution of differentiated mesoangioblast-derived myofibers forming a robust three-dimensional network (Figure 1A,B). The PF hydrogels supported mesoangioblast adhesion and differentiation, as shown by immunofluorescence analyses for myosin heavy chain in spontaneously contracting myofibers (Figure 1C; Additional file 2: movie 1). Under the scanning electron microscope, the differentiated mesoangioblasts were seen to be organized into mature muscle fibers embedded within the PF hydrogels (Figure 1D).

### Polyethylene glycol-fibrinogen scaffold enhances mesoangioblast-mediated regeneration after freeze injury

**Figure 2:**
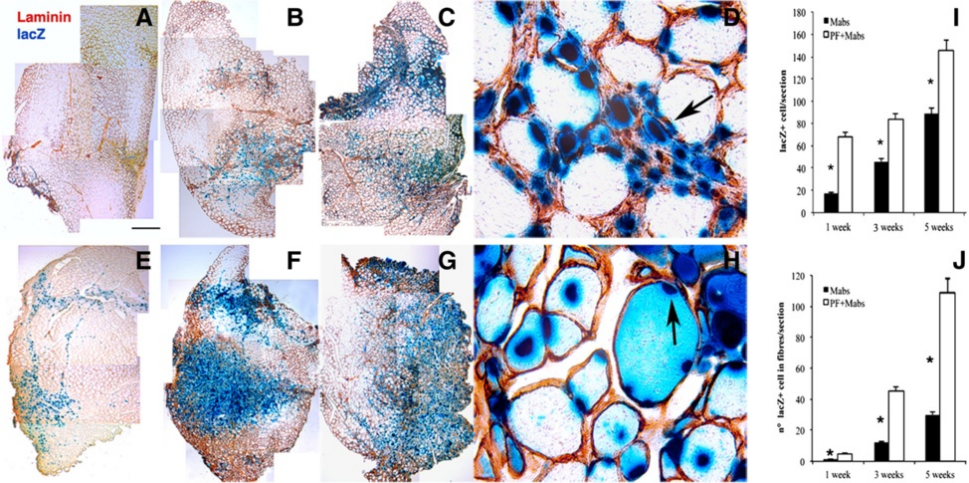
**Long-term engraftment of mesoangioblasts in PBS and of polyethylene glycol-fibrinogen (PF)-embedded mesoangioblasts injected intramuscularly into injured tibialis anterior (TA) muscle.** Sections of injured TA from Rag2 γ-chain null mice after 1, 3, and 5 weeks, respectively of treatment with nuclear (n)lacZ mesoangioblasts in PBS (**A-C**) or in PF (**E-G**) stained with X-Gal (blue) and laminin (red). Histological analyses revealed a higher number of lacZ-positive cells in TA treated with the PF mesoangioblasts, compared with TA treated with the PBS mesoangioblasts. (**H**) High-magnification views of X-Gal and laminin staining showing the localization of lacZ-positive nuclei at the periphery of the host’s mature regenerating muscle fibers (arrow) in TA injected with PF mesoangioblasts. (**D**) The muscle treated with PBS mesoangioblast presented lac-Z positive cells mainly located in the extracellular matrix (arrow) of the TA muscle fibers. The histograms show the number of nlacZ-positive nuclei detected in five randomly selected fields of different, non-adjacent sections (n = 3 mice per group) of X-Gal/laminin-stained TA. (**I,J**) Mean ± SD of nlacZ-positive nuclei (**I**) in the whole TA (cell engraftment evaluation) and (**J**) inside myofibers (cell integration evaluation). Black bars indicate mesoangioblasts injected in PBS, and white bars indicate mesoangioblasts injected in PF, analyzed at 1, 3, and 5 weeks after treatment. Differences were significant (P<0.05) by ANOVA. Scale bar: (A–C,E–G) 500 μm, (**D,H**) 20 μm.

PF hydrogels (8 mg/ml) were used as an *in vivo* carrier for transplantation of nlacZ-mesoangioblasts (3 × 10^5^) (PF-embedded mesoangioblasts) by intra-muscular injection after liquid nitrogen-induced injury to the TA of immunodeficient Rag2 γ-chain null mice [29] (these mice were used to prevent an immune response to β-galactosidase). Mice were killed at 1, 3, and 5 weeks after injection of mesoangioblasts in PF or in PBS, in order to evaluate time-dependent regeneration of the TA muscle. Engraftment of mesoangioblasts in the regenerating muscle was analyzed in TA sections by staining with X-Gal and anti-laminin antibodies that recognize the basal lamina surrounding muscle fibers. Histological analysis showed that the number of lacZ-positive cells was higher in animals treated with PF-embedded mesoangioblasts (Figure 2E-G) compared with controls treated with mesoangioblasts in PBS (Figure 2A-C). At higher magnifications, the mesoangioblasts appeared mainly localized in the ECM of the muscle treated with PBS mesoangioblasts, whereas the PF mesoangioblasts had mainly fused with regenerating fibers. After 3 and 5 weeks after PF mesoangioblasts treatment, most of the lacZ-positive nuclei were centrally located within the fibers, and some of the transplanted cells already occupied a sub-sarcolemmal position in the regenerated fibers (Figure 2H, arrow). By contrast, the injuries treated with PBS mesoangioblasts exhibited significantly fewer nuclei inside newly formed muscle fibers (Figure 2D, arrow); at 5 weeks after injection 110 ± 19 nuclei were scored inside the TA fibers injected with PF mesoangioblasts, compared with 33 ± 6 nuclei in those treated with PBS mesoangioblasts (*P*<0.01) (Figure 2J). Quantitative analysis of nlacZ-positive nuclei per tissue cross-section confirmed the effect of PF in promoting cell engraftment and fusion of mesoangioblasts into the regenerating muscle fibers: at 5 weeks, there were 160 ± 11 nuclei in the PF mesoangioblasts versus 90 ± 8 nuclei in the PBS mesoangioblasts, with the difference being significant (P<0.05) by ANOVA (Figure 2I,J). Moreover, we could detect nlacZ-positive cells adjacent to muscle fibers and expressing Pax7 (muscle satellite cell specific marker), indicating that they were replenishing the satellite-cell pool (see Additional file 3: Figure S3). The number of nlacZ+/Pax7+ cells was also higher in TA muscles injected with the PF mesoangioblasts compared with the PBS mesoangioblasts.

### Polyethylene glycol-fibrinogen enhances survival of mesoangioblasts in freeze injury

**Figure 3:**
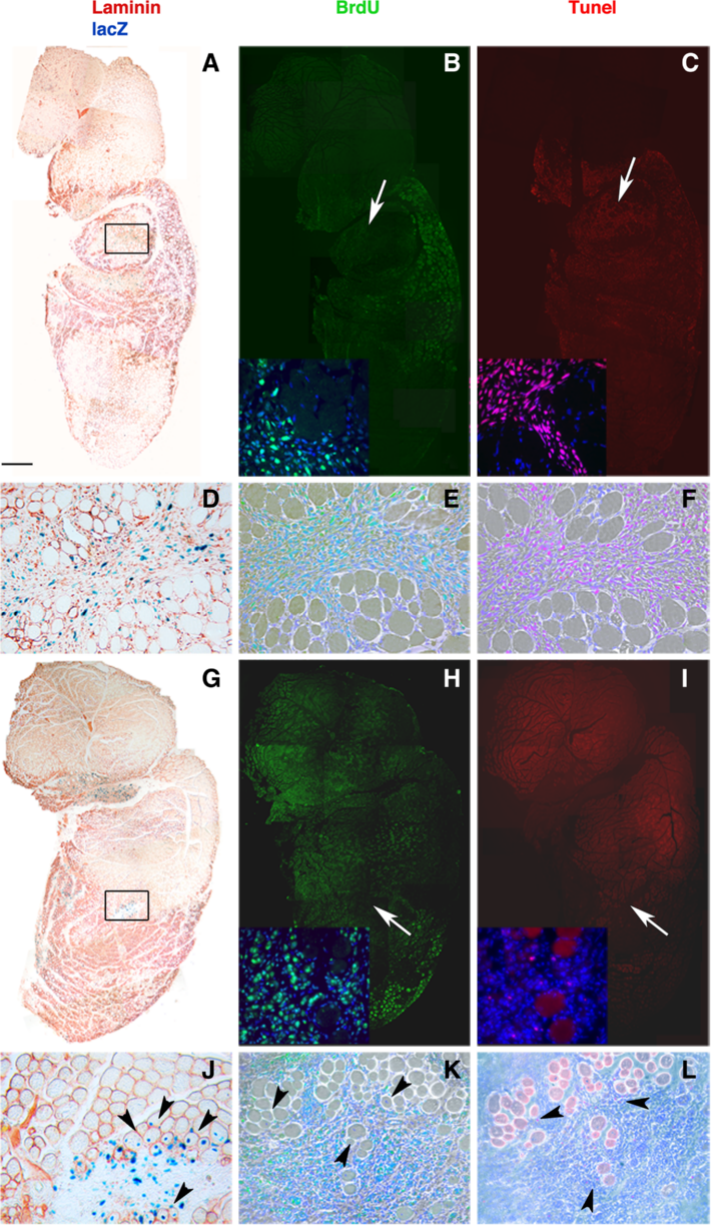
**Survival and proliferation of implanted mesoangioblasts in injured tibialis anterior (TA) muscle of Rag2 γ-chain null mice.** Shown are representative sections 48 hours after intra-muscular injection with nuclear (n)lacZ- mesoangioblasts in (**A**-**F**) PBS or (**G**-**L**) polyethylene glycol-fibrinogen (PF). Graft survival is documented by X-Gal (blue) and laminin (red) staining. The results show higher lacZ-positive cell engraftment in TA treated with the PF mesoangioblasts (**G**,**J**) than with the PBS mesoangioblasts (**A**,**D**). The high-magnification regions (black squares) reveal the localization of lacZ-positive nuclei; these are at the centre of the host’s regenerating muscle fibers (black arrowheads) in the TA muscle treated with the PF mesoangioblasts (**J**), whereas they are mainly located in the extracellular matrix in the TA muscle treated with PBS mesoangioblasts (**D**). Proliferation and apoptosis was assessed by staining with 5-bromo-2-deoxyuridine (BrdU;green) (**B**,**H**) and terminal dUTP nick-end labeling (TUNEL; red) (**C**,**I**); both sets include a nuclear counterstain with 4,6-diamidino-2-phenylindole (DAPI). The decrease in apoptosis in TA sections treated with PF mesoangioblasts (**I**) is striking compared with sections treated with PBS mesoangioblasts (**C**). High-magnification regions (white arrows) of the BrdU- and TUNEL-labelld sections imaged by fluorescence under phase-contrast microscopy show proliferating and apoptotic mesoangioblasts in PBS (**E**,**F**) and PF (**K**,**L**), juxtaposed with the regenerating host muscle fibers. Scale bar: (A,B,C,G,H,I) 500 μm, (D,E,F,J,K,L) 40 μm, (insets) 50 μm.

**Figure 4:**
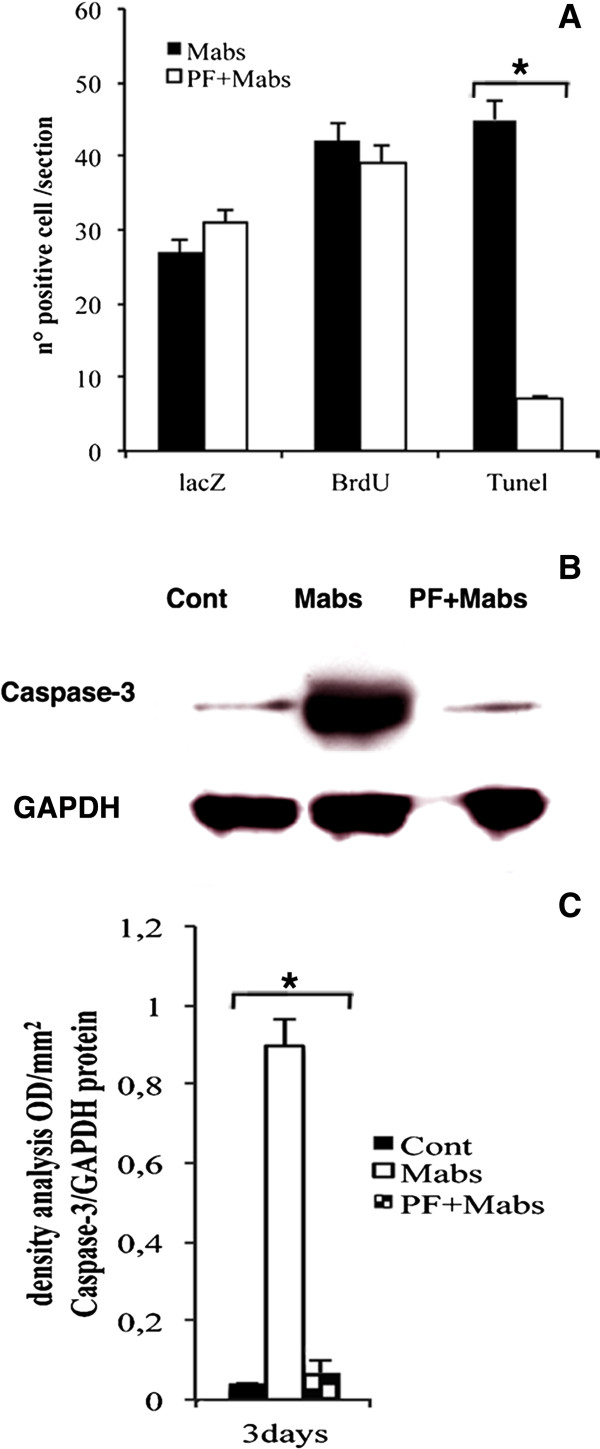
**Quantitative analysis of cell proliferation and apoptosis for mesoangioblasts in PBS and embedded into polyethylene glycol-fibrinogen (PF) injected into injured tibialis anterior (TA) muscle.** (**A**) Number of cells positive for lacZ, bromo-2-deoxyuridine (BrdU) and terminal dUTP nick-end labeling (TUNEL) in five randomly selected, non-adjacent sections of the injured TA from Rag2 γ-chain null mice, 48 hours after injection of nuclear (n)lacZ mesoangioblasts in PBS (black bars) or in PF (white bars). The histogram reveals that the total number of lacZ+ and BrdU+ cells was not significantly different but the number of apoptotic (TUNEL+) cells was reduced by several fold when cells were injected in PF hydrogel (*P<0.01 by ANOVA). (**B**) Western blotting (n = 3, one representative shown in the figure) of cleaved caspase-3 on total protein extracts from the different treatments of injured TA muscle samples from three different Rag2 γ-chain null mice. The data reveal a robust increase in expression of caspase-3 in the TA treated with PBS mesoangioblasts compared with the TA treated with PF mesoangioblasts or sham controls. (**C**) The caspase-3/glyceraldehyde 3-phosphate dehydrogenase (GAPDH) ratio band densitometry data from five different western blots revealed 10-fold higher caspase-3 protein expression level in TA samples injected with PBS mesoangioblasts (white bar) compared with TA samples treated with PF mesoangioblasts (chequered bar). (*P<0.01) between the assessed samples (ANOVA).

The improved mesoangioblast engraftment (Figure 2) associated with the PF hydrogel carrier could be due to reduced cell death and/or enhanced proliferation. To differentiate between these two possibilities, nlacZ-mesoangioblasts (3 × 10^5^) were injected intramuscularly into the injured TA of Rag2 γ-chain null mice that were also treated with the thymidine analog BrdU. The BrdU was incorporated in actively proliferating cells (Figure 3B,E,H,K), which were also assayed by TUNEL nuclear staining that reveals cell death by apoptosis (Figure 3C,F,I,L). Mesoangioblasts delivered using PF hydrogels exhibited a much lower number (7 ± l/section) of apoptotic nlacZ-positive cells (*P*<0.01 by ANOVA (Figure 3I,J), compared with PBS mesoangioblasts (45 ± 4/section) (Figure 3C,F). The mesoangioblast proliferation, as indicated by BrdU incorporation, did not seem to be affected by the PF hydrogel carrier, indicating that protection from apoptosis rather than increased proliferation was the cause of the enhanced engraftment (Figure 4A). To further confirm the anti-apoptotic effect of PF, expression of caspase-3 protein was evaluated. Caspase-3 is a member of the cysteine–aspartic acid protease family that plays a central role in the execution-phase of cell apoptosis. Mice that were killed 48 hours after intra-muscular injection of mesoangioblasts in PBS exhibited much higher levels of activated caspase-3 expression compared with the group injected with PF mesoangioblasts or the untreated sham group (Figure 4B,C).

### Polyethylene glycol-fibrinogen hydrogel improves efficacy of mesoangioblasts in muscular dystrophy

**Figure 5:**
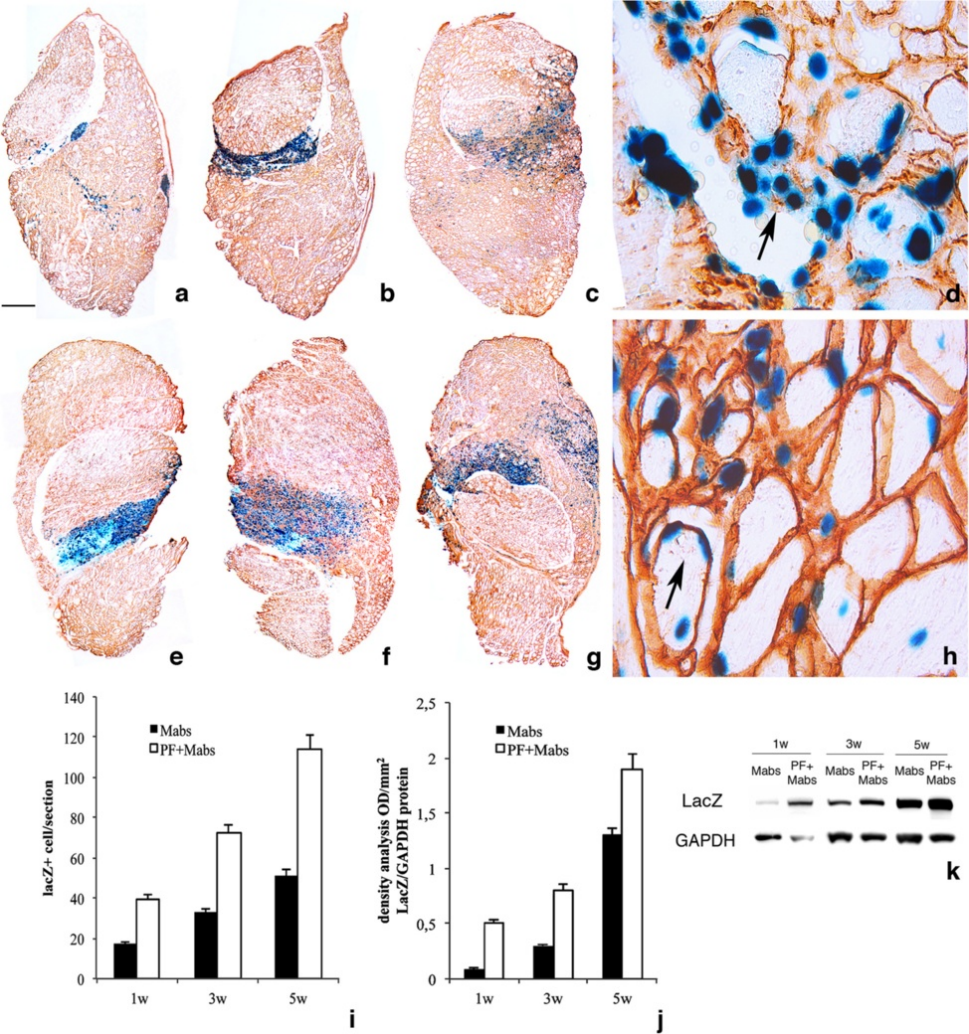
**Survival and engraftment of mesoangioblasts in a dystrophic mouse model.** Shown are different time-point samples (1, 3, and 5 weeks, respectively) of the dystrophic tibialis anterior (TA) muscles from 12-month-old α-sarcoglycan null mice treated (n = 18 per group) with intra-muscular injections of nuclear (n)lacZ mesoangioblasts in **PBS** (**A**-**C**) or polyethylene glycol-fibrinogen (PF) (**E**-**G**). X-Gal staining is shown in blue and laminin immunostaining in red. Histological analysis showed a higher number of lacZ+ cells in the TA muscle treated with the PF mesoangioblasts (**E**-**G**) compared with the PBS mesoangioblasts (**A**-**C**). High magnification of X-Gal and laminin staining reveals an amelioration of the muscle morphology, showing the localization of lacZ-positive nuclei at the periphery of the host’s regenerating muscle fibers (arrow) for the TA injected with the PF mesoangioblasts (**H**), whereas donor nuclei are mainly located in the extracellular matrix (arrow) in the TA treated with PBS mesoangioblasts (**D**). Quantitative analysis of the total number of nlacZ+ nuclei on X-Gal/laminin-stained TA sections reveals higher mesoangioblast engraftment at each time point in the TA muscles treated with PF mesoangioblasts, (**I**) and ameliorated integration of PF mesoangioblasts into host regenerated myofibers (**J**). The number of mesoangioblasts in PBS-injected TA (black bars) and PF-injected TA (white bars) was documented at 1, 3, and 5 weeks after treatment (*P<0.05 by ANOVA test). Counting analysis was performed by scoring lacZ-positive labeled cells under a phase-contrast microscope (× 40) in five randomly selected fields of different non-adjacent sections for three mice per group. (**K**) The representative western blots for lacZ in total protein extracts from three different treated dystrophic TA muscles (n = 5, one representative shown in the figure) show the progressive increase of lacZ expression in the TA muscle treated with PF mesoangioblasts compared with the samples treated with PBS mesoangioblasts. (**L**) Densitometric analysis of the lacZ/GAPDH ratio from five different western blots confirms the histological data analysis, and documents a steady increase in lacZ protein as a function of engraftment time; the influence of the PF carrier on survival and integration of nlacZ-mesoangioblasts is also evident (*P<0.05 by ANOVA test). Scale bar: (A, B, C, E, F, G) 500 μm, (D, H) 20 μm.

The combination of PF hydrogels and mesoangioblasts was also tested as a locally administered cell therapy for repair of dystrophic muscle at an advanced stage of the disease. Although systemic intra-arterial distribution remains the obvious way to target many muscles in diffuse forms of muscular dystrophy, local administration may be a simpler and more efficacious option for localized forms affecting only a few muscles, such as the oculopharyngeal muscular dystrophy (OPMD), and this is already being tested in patients. Accordingly, we administered mesoangioblasts intramuscularly with or without PF in 12-month-old dystrophic mice. These relatively old α-SGKO/SCIDbg mice were chosen because they develop a progressive and more severe muscular dystrophy compared with younger mice or with other strains such as the mdx mouse [21]. Moreover, the sclerosis and reduced microvessel network in these animals impair the efficacy of several alternative treatments [22]. The nlacZ-mesoangioblast grafts (3 × 10^5^ cells) were injected directly into chronically inflamed and sclerotic TA regions typical of the advanced stages of the disease; this represents a more hostile environment for donor cells and, unfortunately, is a common finding in patients with the most severe forms of muscular dystrophy. Immunohistochemistry for lacZ and laminin showed increased engraftment and survival of nlacZ-positive mesoangioblasts when injected with PF (Figure 5E-G) compared with those injected in PBS (Figure 5A-C). Histological analyses of dystrophic muscle 5 weeks after the treatment showed enhanced mesoangioblast integration into regenerating muscle fibers when the PF hydrogel carrier was used (Figure 5H), compared with the PBS carrier (Figure 5D). Laminin staining highlighted that there was better organization of regenerated muscle fibers in the TA treated with the PF mesoangioblasts, with an increased number of nlacZ-positive nucleated fibers, whereas the animals treated with PBS mesoangioblasts exhibited many nlacZ-positive cells still present in the extracellular compartment surrounding the fibers. Quantitative analysis showed a consistent increase in the number of integrated mesoangioblasts inside the regenerated host muscle fibers when they were injected with PF: 27 ± 7 PBS mesoangioblasts per section versus 88 ± 8 PF mesoangioblasts per section (P<0.01) (Figure 5J) and an higher overall number of mesoangioblasts with PF (118 ± 9 PF mesoangioblasts and 51 ± 6 PBS mesoangioblasts) (P<0,05) (Figure 5I), which was also confirmed by quantitative western blotting and relative densitometry (Figure 5K,L).

### Polyethylene glycol-fibrinogen ameliorates mesoangioblast-derived α-SG expression in muscular dystrophy

**Figure 6:**
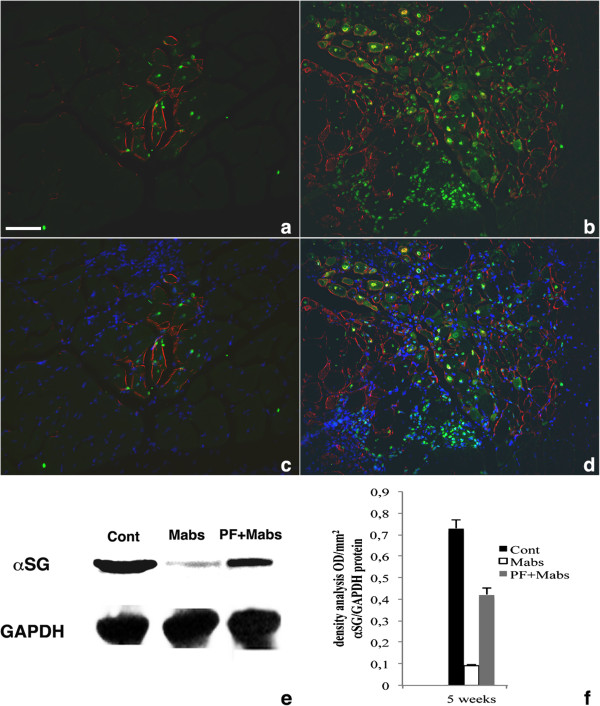
**Expression analysis of α-sarcoglycan (α-SG) on dystrophic tibialis anterior (TA) muscle sections from α-SG null mice.** α-SG immunostaining on TA sections from α-SG null mice 5 weeks after treatment with mesoangioblasts in PBS (**A**,**C**) or with mesoangioblasts in polyethylene glycol-fibrinogen (PF) carrier (**B**, **D**); immunofluorescence of the α-SG is red and that of lacZ is green, with the 4,6-diamidino-2-phenylindole (DAPI) nuclear counterstain being blue (**C**,**D**). The number of α-SG positive fibers was increased with the PF mesoangioblaststreatment and localized in proximity to lacZ-positive engrafted myofibers. (**E**) Western blotting analysis for α-SG in total protein extracts from three different treated dystrophic TA muscles (n = 5, one representative shown in the figure), revealed that the α-SG expression in the dystrophic TA muscle treated with PF mesoangioblasts was higher than that in TA muscle treated with PBS mesoangioblasts, and was closer to the level seen in wild-type controls (Cont). (**F**) The α-SG/glyceraldehyde 3-phosphate dehydrogenase (GAPDH) ratio densitometry analysis from five different western blots revealed higher α-SG protein expression level in the dystrophic TA samples treated with PF mesoangioblasts, reaching a level slightly above 50% of the level seen in wild-type mice (*P<0.05 by ANOVA test of the considered samples). Scale bar: (**A**-**D**) 50 μm.

Sections of TA from dystrophic αSGKO/SCIDbg mice (12 months old) were examined for α-SG expression after treatment with mesoangioblasts (with or without PF carrier). Immunofluorescence at 5 weeks after mesoangioblast injection showed partial recovery of α-SG expression (sarcolemma-associated protein surrounding the myofibers) in dystrophic TA muscles (Figure 6). The expression of α-SG protein was more abundant in sections of TA treated with the PF mesoangioblasts (Figure 6B,D) than with the PBS mesoangioblasts group (Figure 6A,C). Although the α-SG in muscles of αSGKO mice treated with PF mesoangioblasts was not uniformly distributed, the level of protein expression approached those found in the sham controls (wild-type mice treated with PBS injection) (Figure 6E). Quantitative analyses using western blotting densitometry confirmed significantly (*P*<0.05 by ANOVA test) increased expression of α-SG in dystrophic mice treated with PF mesoangioblasts (over 50%) compared with the PBS mesoangioblasts group (12.5%) (Figure 6F).

## Discussion

Various pathological conditions, such as primary or acquired myopathies, can lead to considerable degeneration in and/or loss of skeletal-muscle tissue. Because of its limited capacity for self-repair, reconstruction or regeneration of skeletal muscle often requires exogenous treatments [1]. In particular, skeletal muscle in the advanced stages of muscular diseases cannot regenerate, and the accumulation of fat and connective tissue that replaces the muscle tissue hinders the efficacy of novel treatments such as cell or gene therapy and even drug delivery. Recently, the implantation of an engineered skeletal muscle has been proposed as an alternative strategy for treating advanced-stage muscle pathologies. Engineered-muscle explants offer the possibility of immediate structural repair, prolonged implant survival, and accelerated functional recovery [12].

In this study, we investigated a tissue-engineering approach that is capable of producing enriched donor cell engraftment into skeletal muscle, either after an acute injury or in the more difficult case of advanced-stage muscular dystrophy. We combined a photopolymerizable PF hydrogel carrier with mesoangioblast cells to provide an injectable tissue-engineering treatment option. The PF matrix was tested *in vitro* along with a number of other suitable injectable hydrogel biomaterials and cell types. Ultimately, it was the combination of myogenic cells and PF hydrogels that produced the most promising *in vitro* results, with a thick tri-dimensional network of differentiated myofibers. The human mesoangioblasts embedded into PF showed good myogenic differentiation. Based on our *in vitro* data, the combination of mesoangioblasts and PF was tested in an acute injury model and in a chronic dystrophic mouse model. Although mesoangioblasts show good engraftment in damaged and dystrophic muscle because of their ability to fuse with regenerating myofibers, the injectable PF carrier significantly enhanced this engraftment, and furthermore the PF-embedded mesoangioblasts were able to partly replenish the muscle satellite-cell niche. This effect was due mainly to the encapsulating and protective environment provided by the PF surrounding the embedded mesoangioblasts. This dense resorbable hydrogel milieu provided immediate and timely protection from host inflammation, preventing apoptosis of the cells, without interfering with cell proliferation or impeding long-term graft survival, both in acutely damaged muscle, and in dystrophic muscle at an advanced stage of the disease. Rossi and colleagues recently reported the effect of a photo-crosslinked hyaluronic acid-based hydrogel (hyaluronic acid-photoinitiator; HA-PI). This biomaterial improved the ability of myogenic precursor cells to restore muscle tissue after ablation, leading to functional recovery of injected cell-derived myofibers and to the repopulation of the satellite-cell niche [17]. Moreover, despite using different hydrogels (HA-PI and PF), the range of elasticity to mimic the skeletal-muscle tissue environment (in our condition 8 mg/ml) for both biomaterials is similar, ranging between 150 and 200 Pa. Therefore, even following different routes in terms of the types of cells transplanted and the hydrogel used as scaffold, the results obtained strongly support the evidence that the skeletal muscle tissue-engineering approach could have important clinical applications in muscle recovery of damaged or dystrophy affected muscles.

## Conclusion

The data described in this work provide demonstration of the improved efficacy of mesoangioblast-mediated cell therapy when cells are injected in a resorbable biomaterial such as PF. This material protects injected cells from the apoptosis they would normally undergo in the inflamed or sclerotic muscle environment that is encountered in acute or chronic pathologies of such tissue. Thus, exploiting the features of PF to promote better mesoangioblast engraftment and muscle regeneration may result in a significant benefit for patients with localized forms of muscular dystrophy or those with acquired disorders that lead to severe damage of skeletal muscles, including hernia, sphincter disorders, and surgical small-muscle ablations.

## Abbreviations

α-SG: Alpha-sarcoglycan; αSGKO: αSG knockout; BrdU: 5-bromo-2-deoxyuridine; BSA: bovine serum albumin; DAPI: 4 6-diamidino-2-phenylindole; ECM: Extracellular matrix; FCS: Fetal calf serum; GAPDH: Glyceraldehyde 3-phosphate dehydrogenase; HRP: Horseradish peroxidase; nlacZ-mesoangioblasts: mesoangioblasts expressing β-galactosidase; MyHC: Myosin heavy chain; OPMD: Oculo Pharyngeal Muscular Dystrophy; PBS: Phosphate-buffered saline: PEG, Polyethylene glycol; PF: PEG-Fibrinogen; SDS-PAGE: dodecyl sulfate polyacrylamide gel electrophoresis; TA: Tibialis anterior muscle; TUNEL: Terminal deoxynucleotidyl transferase dUTP nick-end labeling.

## Competing interests

The authors declare no conflict of interest with the paper.

## Authors’ contributions

GC and CG designed the research and wrote the paper; CF performed the cell proliferation and apoptosis assay and carried out most of the experimental work; AB tested the different PF hydrogel concentrations, KS and DS produced and implemented PF for muscle experiments; MLS and SS performed the histology and tissue staining, SA and FST performed the *in vivo* experiments, SB performed the cell culture and immunostaining; and SMC and DS helped with the data analysis, interpretation, and paper writing. All authors read and approved the final manuscript.

## Supplementary Material

Additional file 1: Figure S1 Comparison between different material and myogenic cells, indicating PF as the best supporting three-dimensional (3D) environment for myogenic precursors muscle differentiation. Fibrin, TG-polyethelene glycol (PEG) and PEG-fibrinogen (PF) hydrogels were also analyzed (at 5 days culture) with different myogenic cells: C2C12 **(A,D,G)**, muscle satellite cells **(B,E,H)** and mesoangioblasts **(C,F,I)**. **(A-F)** Within fibrin and TG-PEG hydrogels, numerous undifferentiated cells (arrowheads) or undifferentiated aggregates (asterisks) and some formed myotubes (arrows) were observed for all cell type tested, while PF **(G-I)** seemed to be a suitable 3D environment, promoting muscle differentiation, as revealed by a complete 3D network of tick-differentiated myofibers with few undifferentiated cells. Insets show enlarged view of undifferentiated (arrowhead) and differentiated (arrows) cells Scale bar: **(A-I)** 200 μm, **(insets)** 50 μm. **Figure S2 Myogenic differentiation in 3D PF environment (8 mg/ml) between mouse and human mesoangioblasts, revealed by myosin heavy chain (MyHC) immunofluorescence. (A)** PF-embedded mouse mesoangioblasts (after 5 days of culture) showed a thick three-dimensional network of MyHC positive (red) fibers composed of large mesoangioblast nuclei (arrows). **(B)** Similar differentiation ability was observed in human mesoangioblasts (at 5 days of culture) embedded in PF (8mg/ml). Nuclei are labeled in blue by 4,6-diamidino-2-phenylindole (DAPI) nuclear counterstaining. **(A, B)**Scale bar: 20 μm. **Figure S3 PF enhances mesoangiobasts derived satellite cell poll replenishment.** Double immunofluorescence for lacZ (green) and Pax7 (red) on section of αsarcoglycan (αS-G) null mice transplanted TA, 5 weeks after injection. PBS injected mesoangioblasts **(A)** and PF-embedded mesoangioblasts **(B)** are identified as satellite cells by co-expression of Pax7 and nuclear (n)lacZ, and appear orange in the merged image (arrows) while endogenous satellite cells appear red (arrowhead). **(C)**The histogram quantifies lacZ+/Pax7+ cells as a percentage of total Pax7-positive cells in five randomly selected fields of different non-adjacent sections for three mice per group (*P<0.05 by ANOVA test). **(A,B)** Scale bar 50μm [23,25,30]. Click here for file

Additional file 2: Movie 1 Contracting myotube in 8 mg/ml polyethelene glycol-fibrinogen (PF). Movie showing a mesoangioblasts derived contracting myotube embedded into PF hydrogel. Click here for file

Additional file 3: Movie 2 Differentiated muscle fibers in 8 mg/ml polyethelene glycol-fibrinogen (PF). Movie revealing different focal plan demonstrating three-dimensional myofiber network produced by PF-embedded mesoangioblasts. Click here for file
