# Supplementary material for: Injectable polyethylene glycol-fibrinogen hydrogel adjuvant improves survival and differentiation of transplanted mesoangioblasts in acute and chronic skeletal-muscle degeneration
Source: Skelet Muscle. 2012 Nov 26;2:24. doi: 10.1186/2044-5040-2-24 (PMC3579757; doi:10.1186/2044-5040-2-24)
Supplement: Additional file 1: Figure S1 — Comparison between different material and myogenic cells, indicating PF as the best supporting three-dimensional (3D) environment for myogenic precursors muscle differentiation. Fibrin, TG-polyethelene glycol (PEG) and PEG-fibrinogen (PF) hydrogels were also analyzed (at 5 days culture) with different myogenic cells: C2C12 (A,D,G), muscle satellite cells (B,E,H) and mesoangioblasts (C,F,I). (A-F) Within fibrin and TG-PEG hydrogels, numerous undifferentiated cells (arrowheads) or undifferentiated aggregates (asterisks) and some formed myotubes (arrows) were observed for all cell type tested, while PF (G-I) seemed to be a suitable 3D environment, promoting muscle differentiation, as revealed by a complete 3D network of tick-differentiated myofibers with few undifferentiated cells. Insets show enlarged view of undifferentiated (arrowhead) and differentiated (arrows) cells Scale bar: (A-I) 200 μm, (insets) 50 μm. Figure S2 Myogenic differentiation in 3D PF environment (8 mg/ml) between mouse and human mesoangioblasts, revealed by myosin heavy chain (MyHC) immunofluorescence. (A) PF-embedded mouse mesoangioblasts (after 5 days of culture) showed a thick three-dimensional network of MyHC positive (red) fibers composed of large mesoangioblast nuclei (arrows). (B) Similar differentiation ability was observed in human mesoangioblasts (at 5 days of culture) embedded in PF (8mg/ml). Nuclei are labeled in blue by 4,6-diamidino-2-phenylindole (DAPI) nuclear counterstaining. (A, B)Scale bar: 20 μm. Figure S3 PF enhances mesoangiobasts derived satellite cell poll replenishment. Double immunofluorescence for lacZ (green) and Pax7 (red) on section of αsarcoglycan (αS-G) null mice transplanted TA, 5 weeks after injection. PBS injected mesoangioblasts (A) and PF-embedded mesoangioblasts (B) are identified as satellite cells by co-expression of Pax7 and nuclear (n)lacZ, and appear orange in the merged image (arrows) while endogenous satellite cells appear red (arrowhead). (C)The histog [file 2044-5040-2-24-S1.pdf]

Additional Figure 1

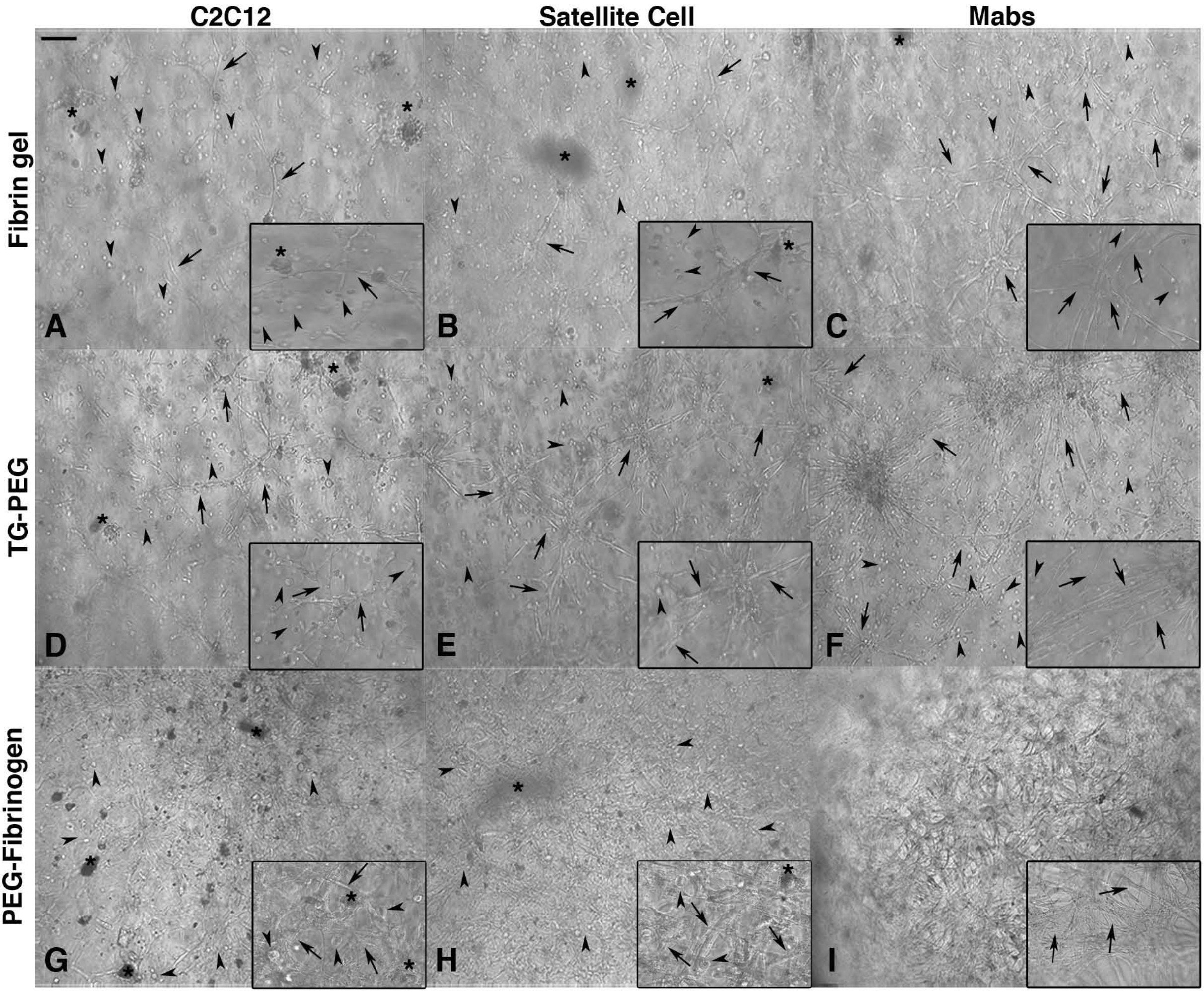

Additional Figure 2

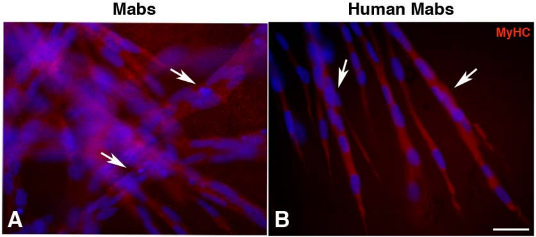

Additional Figure 3

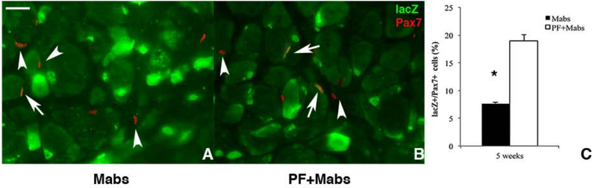

Additional material and methods

Cell culture conditions

C2C12 were cultured at 37°C (5% CO_2) on PETRI dishes with DMEM GlutaMAX (Dulbecco's Modified Eagle Medium with L-glutamine) (GIBCO), supplemented with heat-inactivated 10% FCS (EuroClone), 100 international units ml^{-1} penicillin and 100 mg ml^{-1} streptomycin. For muscle satellite cells preparation, single muscle fibers were isolated from flexor digitorum brevis (FDB), extensor digitorum longus (EDL), and soleus muscles from 2 month old mice [30]. Groups 20 single fibers were seeded onto a Matrigel-coated 35-mm-dish using a single-fiber culture technique. After satellite cells left their parental myofibers and started to proliferate, they were trypsinized and expanded [30]. Muscle satellite cells were cultured at 37°C (5% CO_2) on rat tail collagen type I (Sigma #C3867) coated PETRI dishes (stock solution: 50 ug/ml dissolved in 0.02M acetic acid), with high glucose DMEM GlutaMAX (Dulbecco's Modified Eagle Medium with L-glutamine) (GIBCO) with heat-inactivated 20% FCS (EuroClone), 10% horse serum and 1% chicken embryo extract (CEE), 100 international units ml^{-1} penicillin and 100 mg ml^{-1} streptomycin.

Fibrin hydrogel

Fibrin gels were prepared from Bovine Fibrinogen (MP Biomedicals, Ohio, USA) at 10mg/ml protein concentration in 150mM phosphate buffer saline (PBS), and the solution was sterile filtered using a 0.2µm filter according to Schense and Hubbell [23]. A BCA assay (Pierce Biotechnology, Rockford, IL, USA), which will be described later on (paragraph BCA) was used to determine the protein concentration. Final fibrinogen concentration of 8mg/ml was achieved dissolving fibrinogen in PBS. Fibrinogen polymerization into fibrin was carried out adding human plasma thrombin (Sigma-Aldrich) at a final concentration of 0.376U/ml. The cell were seeded inside the gel mix and than gel and cells were incubated at 37°C, 5% CO_2 to achieve complete polymerization and for cell culture.

Transglutaminase modified PEG hydrogel (TG-PEG)

A 10% w/v stoichiometrically balanced PEG mix stock solution was used, synthetized as described by Erhbar and colleagues [25], containing the 2 substrate peptides TG-MMP-Lys and TG-Gln. Factor XIIIa (200U/ml) was activated with thrombin (20U/ml) for 30 minutes at 37 °C. TG-PEG polymerization was made by addition to the gel of thrombin-activated factor XIIIa, also named transglutaminase, which is responsible for the cross-linking between n-PEG-Gln and n-PEG-MMP-Lys, resulting in a covalent bond between the amine group of Lysine and the ɣ-carboxamide group of Glutamine. A spare volume was used for incorporation of cells and adhesion molecules (Lys-RGD). The reaction was made in TRIS buffer (50mM, pH 7.6), in presence of 50mM $CaCl_2$ and activated factor XIIIa 10U/ml (final concentration). After the addition of the enzyme, drops of the yet liquid precursor (20µl) were pipetted and sandwiched between two silanized-glass slides, obtained by treatment with SigmaCote (Sigma-Aldrich, Switzerland) in order to get a hydrophobic surface. Prior to gelation, the sandwiched drops contacting only hydrophobic surfaces spread spontaneously to form a disc. A small volume of gel was left in the vial in order to check whether the gelation occurred or not. Once the polymerization was complete the gels were gently detached from the slides using a spatula and pipetting some TRIS buffer to favor the detachment and than incubated at 37°C, 5%CO_2 for cell culture.
